# Numerical Simulation of Non-Uniformly Distributed Corrosion in Reinforced Concrete Cross-Section

**DOI:** 10.3390/ma14143975

**Published:** 2021-07-16

**Authors:** Magdalena German, Jerzy Pamin

**Affiliations:** Chair for Computational Engineering, Faculty of Civil Engineering, Cracow University of Technology, Warszawska 24, 31-155 Cracow, Poland; JPamin@CCE.pk.edu.pl

**Keywords:** reinforced concrete, corrosion of reinforcement, chloride concentration, numerical simulation

## Abstract

Reinforced concrete structures can be strongly damaged by chloride corrosion of reinforcement. Rust accumulated around rebars involves a volumetric expansion, causing cracking of the surrounding concrete. To simulate the corrosion progress, the initiation phase of the corrosion process is first examined, taking into account the phenomena of oxygen and chloride transport as well as the corrosion current flow. This makes it possible to estimate the mass of produced rust, whereby a corrosion level is defined. A combination of three numerical methods is used to solve the coupled problem. The example object of the research is a beam cross-section with four reinforcement bars. The proposed methodology allows one to predict evolving chloride concentration and time to reinforcement depassivation, depending on the reinforcement position and on the location of a point on the bar surface. Moreover, the dependence of the corrosion initiation time on the chloride diffusion coefficient, chloride threshold, and reinforcement cover thickness is examined.

## 1. Introduction

Despite many years of research, reinforcement corrosion is still a very concerning and up-to-date problem. Corrosion deteriorates reinforced concrete (RC) structures and can eliminate them from safe usage. This generates huge expenses considering direct costs of maintenance and indirect costs of bypassing the whole problem. For example, if corrosion attacks a bridge or a viaduct, it is necessary to repair the structure, but also to find another route which takes over the traffic. A major cause of corrosion is chlorides. During the life of a structure, chlorides permeate into concrete. As long as chloride concentration in concrete around reinforcement is below a threshold value, the reinforcement remains to be electro-chemically passive [1,2].

**Figure 1:**
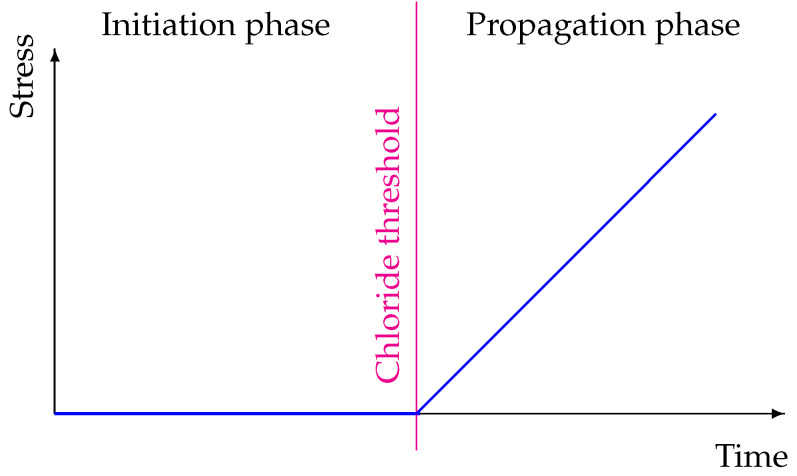
Two-stage Tuutti’s model. Reprint from Ref. [5].

Chloride corrosion is very often represented by the model shown in Figure 1. The stages of the process can be characterized as initiation and propagation phases. Between them, a sudden electro-chemical process takes place. During the initiation phase, chlorides penetrate the concrete cover and accumulate around reinforcement bars. A highly alkaline pore solution provides a passive layer on the reinforcement surface, which protects steel from corroding. However, with time, the pH of concrete decreases, for instance due to the presence of chloride ions. When the pH of pore solution drops approximately below 11 and chloride concentration is above a threshold value, the passive layer is decomposed [1]. An electro-chemical cell is formed and, due to the lack of natural anti-corrosion protection, an electric current starts to flow [3]. The current induces the production of rust and the propagation phase begins. A growing amount of corrosion products generates stresses appearing in the element. In the end, the corrosion process can lead to cracking, splitting, delamination, loss of strength, and general failure of the element [2,4].

The corrosion initiation phase is broadly investigated in laboratory experiments. The authors [6,7,8,9,10,11,12,13,14,15] present experimental results, although each study refers to a different aspect of the initiation phase. In [6], the diffusivity of concrete with different admixtures is investigated. It appears that the calcium content is the most important factor of decreasing the concrete permeability. In [7], a new test for chloride detection and determination of the chloride threshold value is proposed. In [8], autonomous healing of cracks by high and low viscosity polyurethane is presented. The crack healing by viscous polyurethane causes a partial barrier against rapid ingress of chlorides through the crack. The research presented in [9] focuses on the possibility of testing the chloride inflow by using an NaCl solution instead of seawater when assessing the performance of structures in marine conditions. The results of the accelerated NaCl test of corrosion in a specimen made using mortar mixed with aerobic microorganisms are presented in [11]. The experiment shows that metabolic activities of microorganisms results in higher resistance against corrosion, especially effective in the case of cracked concrete. On the other hand, the experimental results presented in [10] show that concrete parameters are not the only ones governing the time to depassivation. The steel surface nanocrystalization achieved through a wire-brushing process produces a layer which significantly improves the resistance to chloride-caused corrosion. In [16,17], the analytical models of corrosion are validated by in situ research. In both models, chloride penetration into non-saturated concrete is not represented exclusively by a diffusion transport mechanism, which will be explained in the next section.

In [13], an experiment of accelerated corrosion is presented. According to experimental results, a numerical model based on the Discrete Element Method is proposed to calculate cracking of mortar due to time-dependent non-uniform circumferential corrosion. In [14], an extensive series of tests which examine the influence of chloride threshold on depassivation of reinforcement is presented. The experiment includes different concrete compositions and rebar orientations. A very interesting experiment is presented in [18]. The X-ray CT technique is applied to reconstruct the meso- and micro-structure of concrete as well as the chloride distribution in a specimen. In [19], an extension of the classical Tuutti’s model is presented. A new model, considering a phase between initiation and propagation, is based on many field and laboratory observations. Another accelerated test of chloride-induced corrosion is presented in [15]. Based on the test results, an estimation of the service life of an RC structure is proposed.

The numerical models and simulations of non-uniform corrosion can be found in [12,20,21]. In [20], chloride transport is modeled as governed by diffusion and migration. The chloride binding is calculated with Langmuir isotherm; however, the relation between the mass of rust and the corrosion current is linear. The criterion for depassivation is expressed in terms of corrosion current value instead of a commonly used chloride threshold. In [21], the finite element simulation of chloride ingress is validated by some experimental data. The model considers heat, moisture, and chloride transport in concrete; however, the chloride transport is assumed to be an effect of diffusion only. The simulation presents the parametric studies of chloride ingress, including marine conditions. In [12], a comprehensive corrosion analysis is presented. The detrimental action of rust is introduced as a corrosive volumetric strain rate tensor; moreover, the calculation of the mass of rust causing cracking considers the transport of corrosion products in the interface transition zone. Additionally, the analytical model is verified by an accelerated laboratory test and numerically simulated. As mentioned above, for a proper description of the initiation phase of chloride corrosion, the analysis of the transport of moisture, oxygen, and chlorides in concrete needs to be considered. In the case of water transport in aged concrete, diffusion is often assumed as the only mechanism governing the problem [22,23,24]. However, other models of capillary water sorption have also been analyzed in [2,25,26]. In the present paper, the averaged concentration of pore water is assumed; thus, both oxygen and chloride transport models become moisture independent, and the water transport is not simulated.

Another problem to be considered is the oxygen transport, as the cathodic reaction is controlled by oxygen concentration $C_{ox}$. In [2], the oxygen transport is analyzed as diffusion-driven, while, in the papers of Ozbolt et al. [3,22,27,28], it is described as a convective-diffusive problem. In aged concrete, the oxygen concentration can be assumed as uniformly distributed and equal to a boundary value, thus the oxygen transport analysis can be neglected.

Although many researchers have analyzed the corrosion phenomena, there are still more unknowns than certainties. For example, in [29], calculations using input data derived from both laboratory specimens and from structures illustrate a poor predictive capacity of known models. An advanced chloride ingress model does not necessarily improve the prediction of the time to corrosion significantly because the phenomenon is too complex, and laboratory results are not always representative.

The mechanical effect of chloride corrosion is usually idealized by pressure acting on concrete and uniformly distributed around the reinforcing bar. However, the geometry of rust is rather irregular and rarely uniformly distributed around the reinforcement. Moreover, reinforcement bars do not corrode equally fast in time. Rust generation starts earlier in the rebars placed in the corners of the cross-section than in those placed along the edges. The most common model used for the calculation of the mass of steel consumed in the process is the Faraday’s law, which neglects the protective function of a thick rust layer. This is included for instance in the model presented by Liu [30].

In this paper, a model of concrete beam cross-section reinforced with four rebars is examined. The numerical simulation of the RC element considers oxygen transport and chloride penetration driven by diffusion and migration. When the chloride concentration exceeds a threshold, the corrosion current density is computed. The mass of corrosion products is calculated using the combined model proposed by Balafas and Burgoyne [31]. This makes it possible to assess the so-called corrosion level that can be used to simulate the mechanical consequences of rust production. The experimental and numerical tests of the mechanical response of a corroded RC element are presented for instance in [32,33,34,35,36]. This last aspect is, however, not the subject of this paper.

The novelty of the work is the simulation of non-uniform distribution of corrosion products around the reinforcing bars in the considered beam cross-section. A nonlinear relation between the corrosion current density and the mass of rust is used. Thereby, a more realistic simulation of the corrosion initiation phase is obtained, and it is possible to estimate the time to depassivation depending on the reinforcement position in the cross-section and on the location of a point on the bar surface.

The paper is organized as follows. A detailed description of the corrosion initiation phase and corrosion macro-cell generation is presented in Section 2. In Section 3, the proposed numerical analysis procedure is described. In Section 4, the results of chloride transport and rust production simulation are presented. Section 5 provides some discussion and conclusions.

## 2. Advanced Model of Initiation Phase

In the paper, concrete is considered at the macroscopic scale, with a constant oxygen diffusion coefficient, since an average concentration of pore water is assumed, and the oxygen flux is expressed just as a diffusion driven process:

(1) $$J_{ox}=D_{ox}\nabla C_{ox}$$

where $C_{ox}$—oxygen concentration is expressed in $\mathrm{kg}/m^{3}$; $D_{ox}$—oxygen diffusion coefficient in $m^{2}/s$.

**Figure 2:**
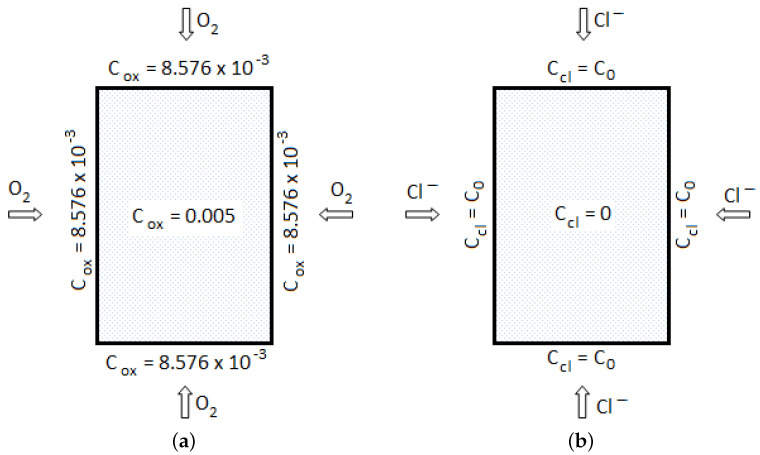
Boundary conditions for calculations of: (**a**) oxygen concentration, (**b**) chloride concentration.

Hence, the equation of mass conservation of oxygen can be written as follows:

(2) $$\frac{\partial C_{ox}}{\partial t}=\nabla \left(D_{ox}\nabla C_{ox}\right)$$

with the initial condition in the domain volume Ω and boundary condition on the domain surface Γ (see Figure 2a) [2,22]:

(3) $$C_{ox}\left(\bar{x},0\right)\;=0.005,\;\;\;\bar{x}\in \Omega$$

(4) $$C_{ox}\left(\bar{x},t\right)\;=8.576\times 10^{-3},\;\;\;\bar{x}\in \Gamma$$

It is assumed for simplicity that the boundary oxygen concentration is constant and uniform.

The main point of the initiation phase analysis is the chloride transport in concrete. For uncracked, unsaturated concrete, in a general case, the total ion flux can be caused by diffusion, migration, advection, and chemical activity of ionic species in the solution. Some authors assume only a diffusion-driven transport [25,37,38]. The chloride diffusion models depend on capillary water, temperature, degree of hydration, or relative humidity. In [2,3,16,22,26,27,28,39], the chloride transport is a result of diffusion and convection. On the other hand, the authors in [17,20,40] assume the chloride transport to be a result of two driving forces–diffusion and migration. The migration term should not be neglected in the description of ionic transport, since ions are charged particles, and this builds up a local electric field [24,40].

In the present paper, the total chloride ion flux $J_{cl}$ is described by the equation:

(5) $$J_{cl}=-D_{cl}\cdot \mathrm{grad}\left(C_{cl}^{f}\right)+\frac{zFD_{cl}}{RT}C_{cl}^{f}\cdot \mathrm{grad}\left(E_{cl}\right)$$

where $D_{cl}$ is the chloride diffusion coefficient, assumed as constant material parameter (in averaged conditions at macroscale) in $m^{2}/s$; $C_{cl}^{f}$ is the free chloride concentration expressed as % of cement mass; *z* is the valence of the chloride ions; *F* is the Faraday constant; *R* is the universal gas constant; *T* is the absolute temperature; and $E_{cl}$ is the local electric potential set up by drifting ions in V.

The local electric potential produced by drifting ions can be determined on the basis of the Poisson equation [24,40]:

(6) $$\nabla^{2}E_{cl}=\frac{zFC_{cl}^{f}}{\varepsilon_{m}}$$

where $\varepsilon_{m}$ is the dielectric constant of the medium (pore water).

During the initiation phase, a part of the chlorides reacts with hydration products and cement. Only the free chlorides cause the corrosion of the reinforcement [2,22,24,37]. The total chloride concentration is a sum of free $C_{cl}^{f}$ and bound $C_{cl}^{b}$ chlorides. Ozbolt et al. [22], as well as Oh and Jang [39], proposed a linear relation between bound and free chlorides. Among the nonlinear forms, there are two models commonly used: Freundlich or Langmuir isotherms [2,24,37]. The Freundlich isotherm, implemented in the numerical model presented in this paper, is a power law between bound and free chloride concentration:

(7) $$C_{cl}^{b}=\alpha {\left(C_{cl}^{f}\right)}^{\beta }$$

where α, β are empirical constants.

Considering this fact, the mass conservation law can be written as:

(8) $$\frac{\partial C_{cl}^{f}}{\partial t}+\frac{\partial C_{cl}^{b}}{\partial t}=\nabla J_{cl}$$

which leads to:

(9) $$\frac{\partial C_{cl}^{f}}{\partial t}=\frac{1}{1+\alpha \beta {\left(C_{cl}^{f}\right)}^{\beta -1}}\nabla J_{cl}$$

with initial and boundary conditions specified as (see Figure 2b):

(10) $$C_{cl}\left(\bar{x},0\right)\;=0,\;\;\;\bar{x}\in \Omega$$

(11) $$C_{cl}\left(\bar{x},t\right)\;=C_{0},\;\;\;\bar{x}\in \Gamma$$

It is assumed for simplicity that the boundary chloride concentration is constant and uniform.

This model has also been investigated earlier and gave satisfactory results when compared to experimental data [5]. This procedure is only applicable in the case of uncracked concrete. The cracked concrete is not a homogeneous porous material, and the rate of diffusion and migration of chloride ions towards the reinforcement additionally depends on crack characteristics.

The time of depassivation is strongly dependent on the threshold chloride concentration. The threshold chloride concentration can depend on several parameters (the condition of steel/concrete interface; the binding capacity of concrete, the pH level of pore solution; exposure conditions such as the source and type of chloride contamination, temperature, and moisture content) and is usually expressed as a percentage of cement mass. The reported values range from 0.17 to 2.5% [2]; however, it is usually assumed that, for reinforced concrete composed with ordinary Portland cement, the chloride threshold is 0.4% of cement mass.

**Figure 3:**
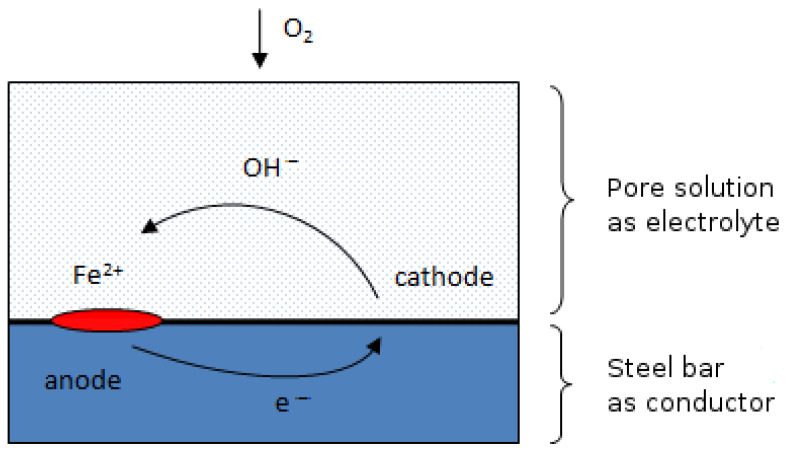
Corrosion cell model in the propagation phase.

Once the chlorides’ concentration equals the threshold value, the corrosion propagation phase begins. The rebar passive layer is decomposed, the corrosion cell is formed, and anode/cathode areas are distinguished. The location of passive layer decomposition is random and depends on the porosity of concrete, properties of pore solution, and mechanical influences. Iron is oxidized to ferrous ions at the anode, and the oxygen is reduced releasing hydroxide ions at the cathode. The pore solution is an electrolyte and the steel rebar is a conductor. The macro-cell model is illustrated in Figure 3. The description of the electro-chemical process occurring in the propagation phase can be found in [1,2,3,25]. In [2,3], the corrosion current is calculated as a result of the electro-chemical process, considering Butler–Volmer kinetics. On the other hand, in [25,31,41], the corrosion current is expressed as an empirical function of chloride concentration, temperature, concrete resistivity, and time.

The current flow causes the polarization of cathode and anode areas. According to the Butler–Volmer kinetics, the anodic reaction is subject to activation polarization, while the reaction at the cathode is subject to activation and concentration polarization [2,42,43], according to the following expressions:

(12) $$i_{a}=i_{oa}\exp\left(2.3\frac{E_{a}^{0}-E_{corr}}{\beta_{a}}\right)\;\;\;\;\;\;i_{c}=i_{oc}\frac{C_{ox}}{C_{ox}^{s}}\exp\left(2.3\frac{E_{corr}-E_{c}^{0}}{\beta_{c}}\right)$$

where $i_{a}$—current density of iron oxidation reaction (at anode) in $A/m^{2}$, $i_{c}$—current density of oxygen reduction reaction (at cathode) in $A/m^{2}$, $i_{oa}$—exchange current density for iron dissolution, $1.87\times \;10^{-4}\;A/m^{2}$, $i_{oc}$—exchange current density for cathodic reaction, $6.25\times \;10^{-6}\;A/m^{2}$, $E_{a}^{0}$—standard potential in anodic reaction, –0.780 V, $E_{c}^{0}$—standard potential in cathodic reaction, 0.160 V, $E_{corr}$—corrosion potential in V, $\beta_{a}$—activation Tafel slope for anodic reaction, 0.06, $\beta_{c}$—activation Tafel slope for cathodic reaction, 0.16, $C_{ox}$—dissolved oxygen concentration at steel surface, and $C_{ox}^{s}$—dissolved oxygen concentration at the external concrete surface.

The supply of oxygen and water is essential for the progress of steel corrosion; however, water does not directly control the process. The oxygen flux at the cathode surface $j_{c}^{ox}$ can be expressed in terms of electric current density $i_{c}$ [2,3]:

(13) $$j_{c}^{ox}=D_{ox}\frac{\partial C_{ox}}{\partial n}{|}_{cathode}\cdot n=-\frac{1}{F\omega_{c}}i_{c}$$

where n is outward normal to the surface, and $\omega_{c}$ is the ionic valence of the ion consumed in the cathodic reaction.

On the other hand, the oxygen consumption at the anode $j_{a}^{ox}$ for $4\mathrm{Fe}{\left(\mathrm{OH}\right)}_{3}$ assumed as a main corrosion product is given by [2,3]:

(14) $$j_{a}^{ox}=D_{ox}\frac{\partial C_{ox}}{\partial n}{|}_{anode}\cdot n=-\frac{32}{359.4}\frac{1}{F\omega_{a}}i_{a}$$

where $\omega_{a}$ is the ionic valence of the ion produced in the cathodic reaction.

Equation (12) can be used to define the electro-chemical conditions at the steel-concrete interface. The equation of electrical charge conservation combined with Ohm’s law reads:

(15) $$\nabla i=-\sigma \nabla^{2}E=0$$

where i—current density vector; σ—electrical conductivity of concrete, assumed constant, uniformly distributed, equal to 0.75 $\times \;10^{-3}\;1/\Omega$m; and *E*—electrical potential.

For the boundary conditions calculated from Equation (12), the solution of Equation (15) determines the distribution of the electric potential and corrosion current.

**Figure 4:**
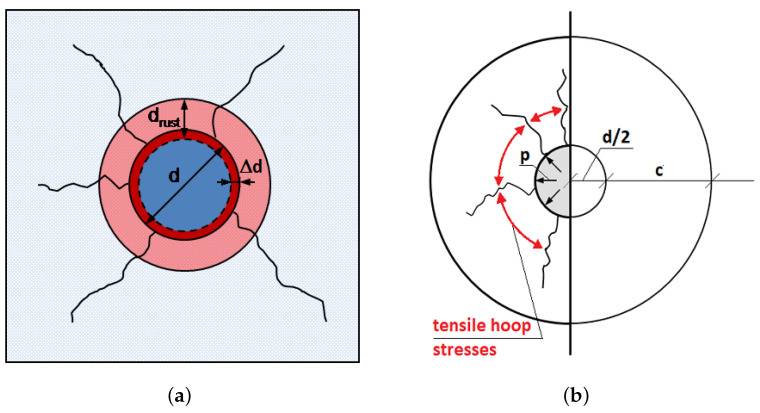
Rust volumetric expansion in propagation phase: (**a**) idealization of steel consumption and rust expansion, *d*—rebar diameter, Δd—loss of rebar diameter due to steel consumption, $d_{rust}$—rust layer thickness, (**b**) illustration of radial pressure and circumferential tensile stresses, *p*—pressure generated by rust expansion, *c*—concrete cover thickness. Cite from Ref. [5].

Oxides created during the iron oxidation process can have even six times larger volume than iron. When the volume of all corrosion products takes too much space, hoop stresses exceed the concrete tensile strength and radial cracks appear (Figure 4). This eventually leads to cracks parallel to the reinforcement bar and visible at the concrete surface.

In view of the fact that it is not possible to reliably define the rust structure, mass and density of rust can be introduced by the following relations:

(16) $$r_{m}\;M_{r}=M_{s}\;\;\;\;\;\;\;\;\;\gamma \;\rho_{r}=\rho_{s}$$

where $r_{m}$—iron-to-rust molecular weight ratio, with typical values 0.523 (for $\mathrm{Fe}{\left(\mathrm{OH}\right)}_{3}$) or 0.622 (for $\mathrm{Fe}{\left(\mathrm{OH}\right)}_{2}$); $M_{r}$—mass of rust in kg/m; $M_{s}$—mass of steel consumed in the process in kg/m; γ—parameter with a value usually ranging 2–4; $\rho_{r}$—rust density; $\rho_{s}$–steel density, 7890 $\mathrm{kg}/m^{3}$.

The most common model used for the calculation of the mass of steel consumed in the process is Faraday’s law. It was used in [2,3,4,25,44] and takes the form:

(17) $$\frac{dM_{s,Faraday}}{dt}=\frac{m_{a}\pi d}{zF}i_{corr}=A_{Faraday}\;i_{corr}$$

where $m_{a}$—atomic weight of dissolved ions (for Fe $m_{a}$ = 55.85 g/mole), *z*—valence of ions, assumed to be equal to 2, $i_{corr}$—corrosion current density, *d*—rebar diameter.

Considering Equation (16), the mass of rust produced in the process can be expressed as:

(18) $$M_{r,Faraday}=\frac{A_{Faraday}}{r_{m}}\;i_{corr}\;\Delta t$$

where Δt—time of propagation phase in seconds.

However, as the rust layer thickens, the iron ionic diffusion distance increases, so the diffusion rate goes down and the rate of rust production decreases. In [30], Liu proposed an alternative formula, assuming a variable rate of rust production with time, also used in [4]:

(19) $$\frac{dM_{r,Liu}}{dt}=\frac{\kappa_{p}}{M_{r}}\;\;\;\;\;\;\;\kappa_{p}=\frac{k}{r_{m}}\pi D\;i_{corr}=\frac{A_{Liu}}{r_{m}}\;i_{corr}$$

where *k* is the empirical constant.

The parameter *k* is a coefficient that allows for a reduction of corrosion rate with rust thickness and usually is evaluated by fitting the model to published experimental data [31]. In the present paper, it is equal to $2.48614\times 10^{-6}$, the rebar diameter is expressed in meters, while the corrosion current in $A/m^{2}$.

Both models of rust production have disadvantages. The Faraday model does not include the presence of the gradually thickening protective rust layer. On the other hand, the formula proposed by Liu [30] gives unrealistic corrosion rates for short times. Balafas and Burgoyne proposed in [31] a combined rule, assuming that initially the corrosion rate is constant, following Faraday’s law, and later it is evaluated using Liu’s expression. The turning point is the moment when the rates of rust production calculated with both models are equal:

(20) $$M_{r}=\left\{\begin{array}{cc} \frac{A_{Faraday}}{r_{m}}i_{corr}\;\Delta t & \mathrm{for}\;\frac{dM_{r,Faraday}}{dt}\leq \frac{dM_{r,Liu}}{dt}\;\\ \sqrt{2\cdot \frac{A_{Liu}}{r_{m}}\cdot i_{corr}\cdot \Delta t} & \mathrm{for}\;\frac{dM_{r,Faraday}}{dt}>\frac{dM_{r,Liu}}{dt} \end{array}\right.$$

**Figure 5:**
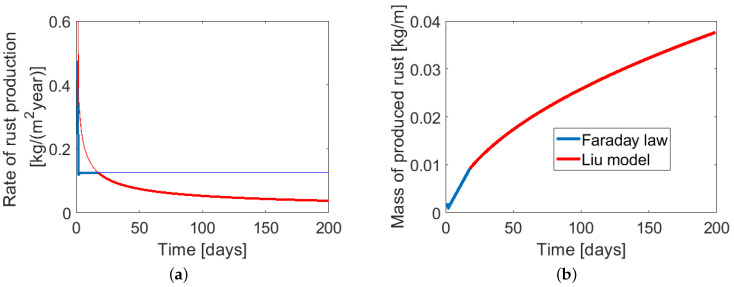
Diagrams for the combined model used for computation of rust mass, proposed in [31]: (**a**) rate of rust production, (**b**) mass of rust produced.

The illustration of the Balafas and Burgoyne combined model is presented in Figure 5. In the present paper, the calculations of $M_{r}$ are performed according to this model.

It must be pointed out that, due to varying concrete cover carbonation and content of chloride ions around the bar, the geometry of rust is rather irregular, i.e., rust is rarely uniformly distributed around the reinforcement. This means that the corrosion starts at the first point of depassivation, in other words at the point subjected to the highest chloride concentration, and then it propagates the way the depassivation changes.

## 3. Analysis Scheme and Numerical Model

**Figure 6:**
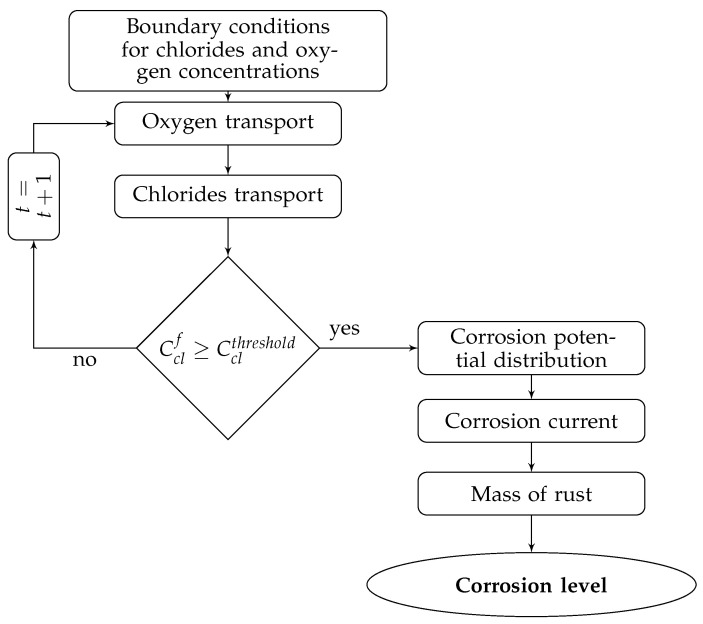
Calculation procedure.

The calculations presented in this paper are performed for a beam according to the procedure presented in Figure 6 and include the determination of chlorides’ concentration, the corrosion current, and volumetric expansion of rust, described by a corrosion level. For this purpose, a Matlab script is written considering the mathematical model presented in Section 2. 

**Figure 7:**
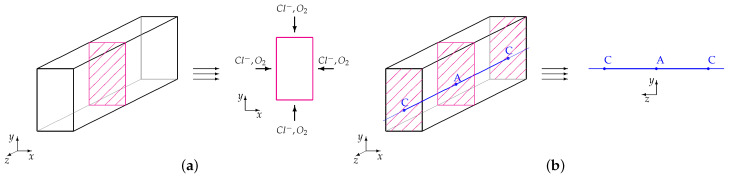
Configurations for calculations of: (**a**) concentration; (**b**) current (C—cathode, A—anode). Cite from Ref. [5].

Since all material parameters are constant and uniformly distributed, one 2D cross-section can be analyzed for concentration calculations. The configuration for oxygen and chloride concentration calculations is presented in Figure 7a.

As the electric current flows through the rebar, the current and potential calculations should be performed in a configuration along the reinforcement axis. In the analysis, it is assumed that one specified beam cross-section is related to anode, and another one is related to cathode. With this assumption, the boundary conditions for the potential distribution can be calculated for specified nodes. The potential distribution and corrosion current are calculated in 1D configuration presented in Figure 7b.

**Figure 8:**
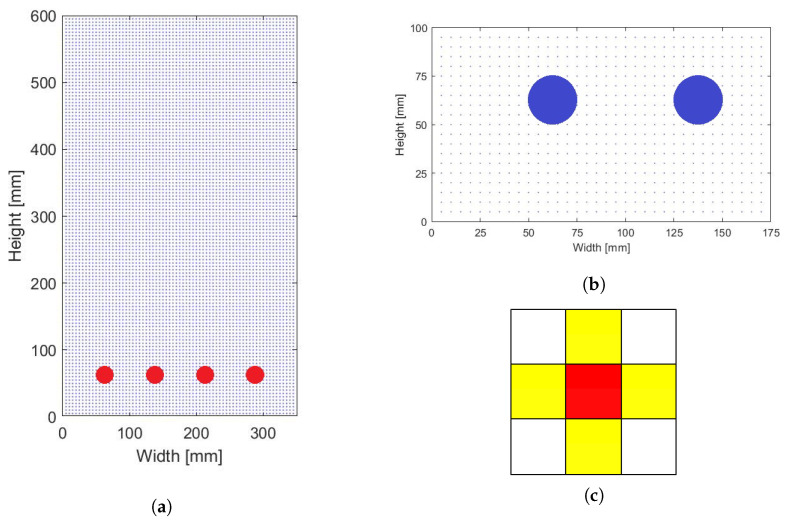
Configuration for concentration calculations: (**a**) general configuration of CA, each node is a cell, (**b**) close-up of analyzed region (half of cross-section width). The distance between cells is Δx = 5 mm, (**c**) von Neumann neighborhood, red is analyzed cell, yellow are neighbors.

The concentrations are computed using cellular automata (CA). CA are mathematical idealizations of physical systems in which space and time are discrete and taken from a finite set of discrete values. CA are a very efficient tool in concentration calculations. They have been successfully used in [37,38,45]. Figure 8 shows the CA discretization adopted for the considered problem. The model based on CA has been more thoroughly presented in [5].

Equations (2) and (9) can be effectively simulated by adopting a von Neumann neighborhood with radius equal to 1 (Figure 8), which means the concentration calculated in the closest neighbors affects the value of concentration in the considered cell. The rule of evolution is expressed as [38,45]:

(21) $$C_{i}^{k}=\varphi_{0}\cdot C_{i}^{k-1}+\sum_{j=1}^{n}\left(\varphi_{j}^{-}\cdot C_{i-1,j}^{k-1}+\varphi_{j}^{+}\cdot C_{i+1,j}^{k-1}\right)$$

where $C_{i}^{k}$—concentration of substance in cell *i* at time *k*, *n*—number of dimensions (*n* = 2).

The values of the evolutionary coefficients must satisfy the following normalization rule, required by the mass conservation law [38,45]:

(22) $$\varphi_{0}+\sum_{j=1}^{n}\left(\varphi_{j}^{-}+\varphi_{j}^{+}\right)=1$$

To ensure the stability of the numerical procedure, the relative time step measure should be maximum 0.5 [37,38,45]:

(23) $$\frac{\Delta t\cdot D_{i}}{\Delta x^{2}}\leq 0.5$$

where Δt—time step, $D_{i}$—diffusion coefficient for a particular substance, and Δx—grid dimension.

The diffusion potential of chlorides used in Equation (9) is calculated in the 2D cross-section configuration (Figure 7a). The Poisson Equation (6) is solved using the finite difference method (FDM), with central difference expressions and Δx = 5 mm. As chlorides’ concentration changes in every time step, the potential needs to be recalculated for the new data. The configuration of nodes is similar to these presenting cells in Figure 8. The potential calculations are repeated in a loop, until the convergence criterion (relative error based on the maximum difference between two iterations smaller than $10^{-6}$) is satisfied.

The chlorides and oxygen concentrations are calculated in a loop until the free chloride threshold value is reached (see Figure 6). After reaching the chloride threshold, the electric potential distribution is calculated with respect to the current oxygen concentration. Equation (15) is solved using the finite element method (FEM) for a 1D problem with Lagrange shape functions. The distribution is analyzed for the section of a rebar between the anode and cathode. The boundary conditions for the potential at the anode and cathode sites are calculated from Equation (12). For simplicity, linear interpolation between the nodal values is used.

The mass of rust is calculated on the basis of expressions presented in Section 2. The corrosion action that can further be used in mechanical calculations is a key result of this analysis.

The mass of rust determined as a result of calculations is expressed as corrosion level $L_{corr}$, understood as the loss of weight related to initial weight of a rebar, according to:

(24) $$L_{corr}=\frac{r_{m}M_{r}}{\rho_{s}A_{rebar}}$$

where $A_{rebar}$—reinforcement bar area.

## 4. Chloride Concentration Analysis

The free chloride and oxygen concentrations are calculated according to the numerical procedure presented in Section 3. The calculations are made for the cross-section of dimensions 350 mm × 600 mm with grid dimension Δx = 5 mm and time step Δt equal to one day. The cross-section is reinforced with four 25 mm-diameter bars. The simulation is performed for the concrete cover of 50 mm.

In the simulation, the material behavior is governed by the diffusion coefficient which is assumed constant during the calculations since concrete aging is neglected. The model presented in the paper has previously been analyzed in [5] and confronted with the experimental results available in the literature [46,47]. The simulation presented in [5] revealed that the proposed model considering diffusion and migration gave accurate results for the most crucial issue of chlorides concentration, which seems to be the time needed to reach the threshold value (i.e., the time to rebar depassivation). However, the determination of the value of boundary concentration can be a problem. To establish the boundary condition for chlorides, at least one experimental profile should be available. As was presented in [5], it is possible to obtain the chloride profile using the presented model after, for instance, five years, knowing the data of the experiment after 0.5 years of exposure. In addition, the chloride threshold value is an individual parameter of each concrete mix, so there is no unique value that can be used in simulations. This is another issue that can be solved only by experimental examination.

In the present simulation, the parameters have been assumed for uncracked concrete. The mass of cement is 300 $\mathrm{kg}/m^{3}$, the chloride diffusion coefficient is 5 × $10^{-12}\;\;m/s^{2}$, and the chloride boundary concentration $C_{0}$ is assumed to be 2.0% of mass of cement. The α, β parameters in Equation (7) are 0.82 and 0.5, respectively. These data are taken from [37,39] and previous research of the first author. Although they do not come from a specific experiment, they are representative for a typical concrete mixture.

**Figure 9:**
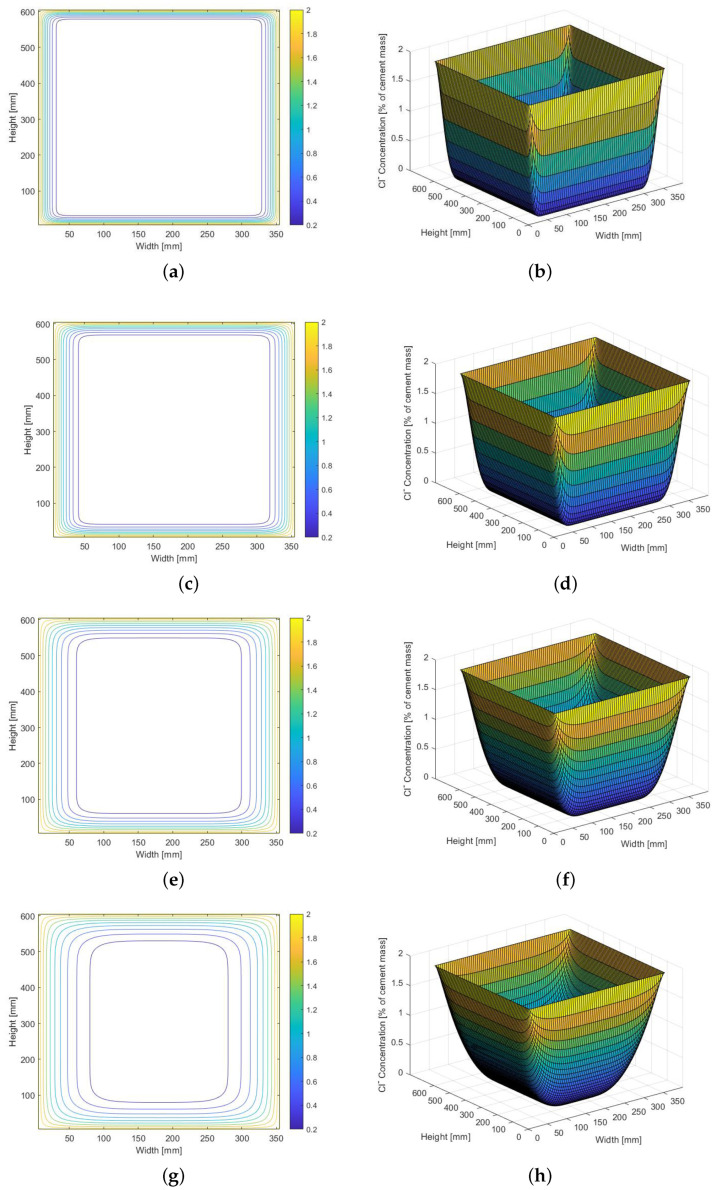
Chloride concentration calculated for cross-section 350 mm wide and 600 mm high, presented as contour plot and in 3D perspective. Concentrations computed after exposure to chlorides for: (**a**,**b**) 1 year, (**c**,**d**) 2 years, (**e**,**f**) 5 years, and (**g**,**h**) 10 years.

The free chloride concentration calculated with the diffusion–migration model are shown in Figure 9. The concentration is expressed as a percentage of cement mass. The simulation has been performed to monitor the chloride concentration change within 20 years of exposure.

**Figure 10:**
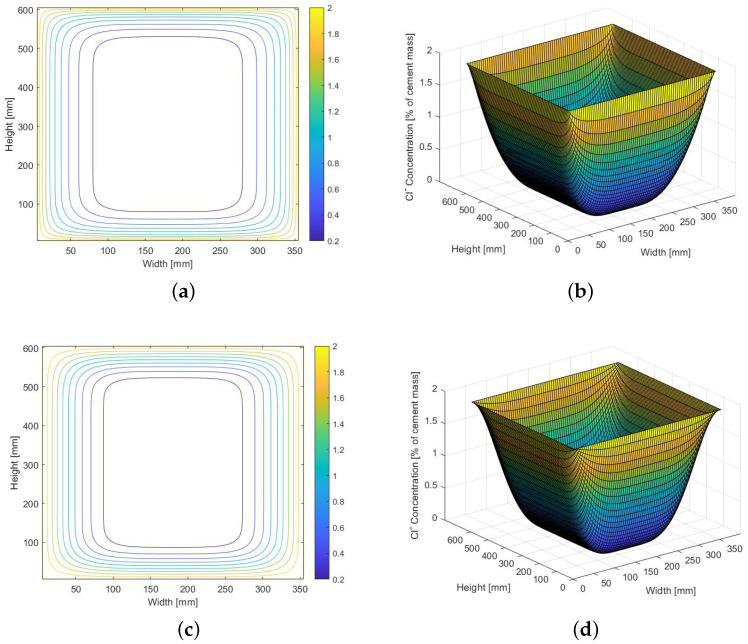
Chloride concentration calculated after 10 years of exposure for cross-section 350 mm wide and 600 mm high, presented as contour plot and in 3D perspective. Concentrations computed using: (**a**,**b**) diffusion-migration model; (**c**,**d**) diffusion model.

A comparison of the results obtained for the model incorporating both diffusion and migration, and the diffusion-based model is presented in Figure 10. The free chloride concentrations are calculated after 10 years of exposure to chlorides. It can be noticed that the concentrations calculated in the second case are higher near the boundary of the domain than those calculated as a result of diffusion and migration.

**Figure 11:**
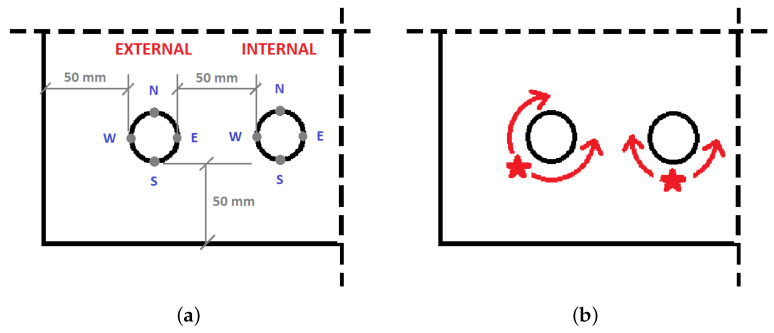
Process of depassivation regarding external and internal rebars: (**a**) points at which depassivation are monitored; (**b**) propagation of depassivation.

Figure 11a presents points at which depassivation is monitored. The process of depassivation has been distinguished for an external and internal rebar. The times to depassivation obtained during the simulation of the 20-year-long process were calculated for three chloride threshold values assumed: 0.3%, 0.35%, and 0.4% of cement mass. The times of depassivation of particular points on the rebar surface for the chloride threshold equal to 0.3% of cement mass are set in Table 1. It can be noticed that depassivation of the external rebar begins at the southwest (SW) point after 3.49 years. After 8.61 years, the whole external rebar surface becomes electro-chemically active. The depassivation of the internal rebar starts at the SW point as well, after 5.38 years. After 14.73 years of exposure, the whole surface of internal rebar is depassivated.

In the case of external rebar, the times monitored at particular points around the rebar are significantly different, and it is a huge mistake to assume uniform corrosion when the mechanical response of concrete is analyzed. However, there is visible symmetry along the SW-NE line. Quite similar results have been obtained in [20]. On the other hand, in the case of internal rebar, the difference in time to depassivation between SW and SE points is just two days, while, between SW and W, it is 4.5 years. Thus, it can be assumed that the depassivation of the internal rebar starts at the S point and propagates symmetrically, just as shown in Figure 11b.

A very similar behavior can be observed when the chloride threshold is equal to 0.35% or 0.4% of the mass of cement. The times of depassivation of particular points on the rebar surface calculated for the threshold value of 0.35% are set in Table 2, while, for 0.4%, are set in Table 3.

For the chloride threshold equal to 0.35% of cement mass, the depassivation of the external rebar starts at the SW point after 3.83 years. After 9.69 years, the whole external rebar surface is depassivated. The passive layer of the internal rebar breaks at SW point, after 6.25 years. After 20 years of exposure to chlorides, the whole surface is depassivated, although the northern points remain passive for over 18 years.

For the chloride threshold equal to 0.4% of cement mass, the depassivation of the external rebar starts at the SW point after 4.19 years. After 10.99 years, the whole external rebar surface is depassivated. The passive layer of the internal rebar breaks at the SW point, after 7.31 years. After 20 years of exposure to chlorides, the northern points on the surface of internal rebar are still passive.

Again, in the case of the external point, the times calculated for the threshold of 0.35% and 0.4% of cement mass, monitored at specified points around the rebar are significantly different, and uniform corrosion should not be assumed in mechanical response considerations. Still, in the case of internal rebar, the difference in time to depassivation between SW and SE points is a couple of days, while, between SW and W, it is a couple of years. Thus, the propagation of depassivation presented in Figure 11b is valid, despite the change of the chloride threshold.

It is worth noticing that, when the internal rebar becomes depassivated at the first point, the external rebar has more than half of its surface depassivated and is already heavily corroding. This could lead to relatively quick spalling of the RC cross-section’s corners; however, this paper does not include mechanical simulation of RC behavior under corrosion. Such analyses were presented in [2,5,44,48,49], yet they considered uniformly distributed corrosion, which in the light of current results was quite a strong assumption.

In the case of external rebar, the increase of chloride threshold from 0.3% to 0.4% of cement mass results in an extension of time to first depassivation by 20%. The time to depassivation of the whole surface of external reinforcement extended by 28%. The most significant increase is observed for internal reinforcement, and the change is equal to 36%, yet, no matter what the chloride threshold is, the rust production at the external reinforcement bar is much more advanced than at the internal one.

In [21], a similar distribution of corrosion products can be observed, and an increase of concrete cover and chloride threshold value causes a nearly linear increase in the time to depassivation. In [21], the times to depassivation are higher than in our work, but the model of chloride ingress used in [21] is different than ours. The other model we compared to is the one presented in [20]. The times to depassivation presented in [20] are shorter than ours. The chloride concentration is calculated using a diffusion–migration model, yet there is a different chloride binding relationship, different depassivation criterion, and different reinforcement diameter that are used. However, the corrosion product distribution is similar to ours, and the parametric studies generally seem to confirm our results.

**Figure 12:**
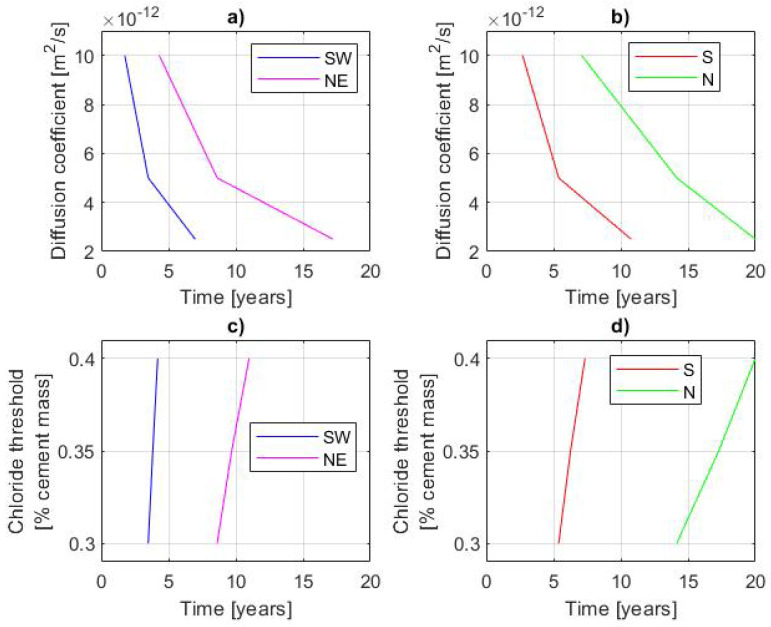
Dependence of initiation time on: (**a**) diffusion coefficient for external rebar; (**b**) diffusion coefficient for internal rebar; (**c**) chloride threshold for external rebar; (**d**) chloride threshold for internal rebar.

**Figure 13:**
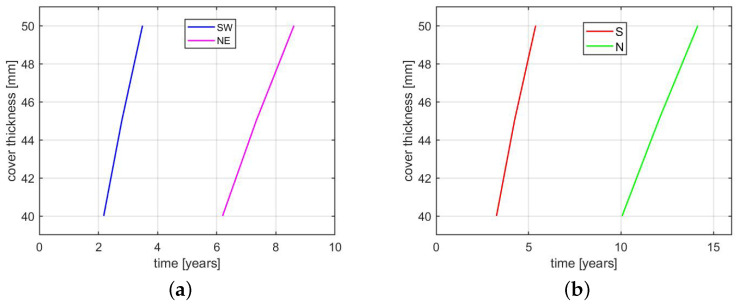
Dependence of initiation time on concrete cover thickness: (**a**) external rebar; (**b**) internal rebar.

Moreover, some parametric studies have been performed. The sensitivity of the initiation time to the diffusion coefficient and concrete cover thickness has been presented in Figure 12 and Figure 13. The influence of the chloride threshold on the initiation time is presented in Figure 12. The dependencies are monitored for the SW and NE points in the case of external rebar (i.e., the first and the last points of depassivation) and the S and N points in the case of internal rebar. The simulation using different diffusion coefficients is performed with the cover thickness of 50 mm, while the analysis with different cover thicknesses is performed with $D_{cl}$ = 5 $\times \;10^{-12}\;m/s^{2}$.

As it can be seen in Figure 12 and Figure 13, the most important parameter governing the initiation time is the diffusion coefficient. A decreasing value of $D_{cl}$ results in a significant increase of the initiation time. This is visible for all points on external and internal rebars, but point SW on the external reinforcement is less sensitive. As far as other parameters (chloride threshold and concrete cover) are concerned, the situation is similar. The initiation time calculated at external point SW is the least sensitive to a change of those parameters, while point N on the internal reinforcement is the most sensitive. It is worth noticing that using concrete cover smaller than 50 mm leads to complete depassivation of both rebars within 10 years of exposure to chlorides.

**Figure 14:**
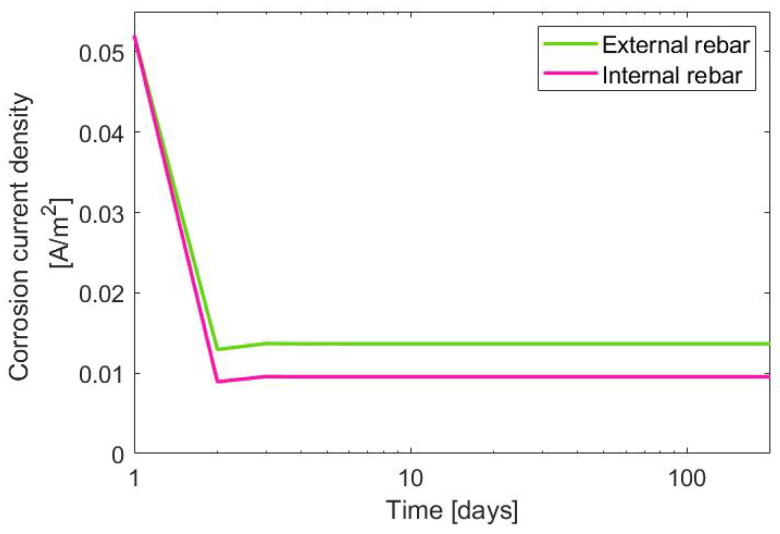
Corrosion current density.

The calculations of the electrical potential provide the information that its change occurs in the first days after depassivation; however, within one month, it stabilizes. A similar behavior is visible in Figure 14, presenting the corrosion current density occurring once the rebar’s passive layer is broken. The horizontal axis is in a logarithmic scale, which allows one to observe the initial change of the current density. After one week from depassivation, the current density stabilizes at the level of $1.4\times 10^{-2}\;A/m^{2}$.

**Figure 15:**
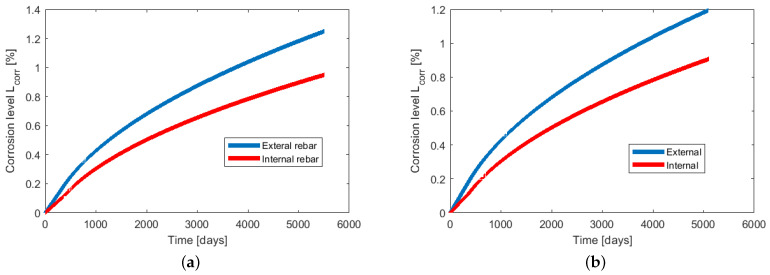
Corrosion level $L_{corr}$ calculated for chloride threshold: (**a**) 0.3% of cement mass; (**b**) 0.4% of cement mass.

In Figure 15, the function of corrosion level $L_{corr}$ in time is presented. $L_{corr}$ is calculated for two chloride threshold values: 0.3% and 0.4% of cement mass. The corrosion level is monitored in the first points of depassivation of both rebars (SW for external and S for internal). The value of chloride threshold does not influence the character of rust production. The only difference observed in Figure 15 is in the time of rust production. Since the time to depassivation is longer, obviously the time of rust production is shorter, and the corrosion level is slightly lower. However, the difference is of minor relevance.

The depassivation of steel reinforcement indicates the end of the corrosion initiation phase and the beginning of the corrosion propagation phase. Rust produced in the process generates stresses in concrete. The results of corrosion level obtained in the proposed way can then be applied as a loading function in further mechanical analysis [5].

## 5. Conclusions

In the paper, the numerical simulation of the initiation phase of chloride corrosion and rust production has been presented. The mechanism of transport of detrimental substances, generation of corrosion cell as well as the propagation of depassivation have been analyzed. The analytical description of chlorides transport considers two driving forces—diffusion and migration—and chloride binding represented by the Freundlich isotherm. The numerical model has been built using the CA. The coupled problems have been solved using FDM and FEM. The analysis is an extension of the previous research, in which rust production was linearly dependent on the corrosion current and uniformly distributed around rebars [5].

The numerical model used for the calculations includes three essential aspects:

- nonlinear relation between free and bound chlorides,
- nonlinear relation between the mass of produced rust and corrosion current,
- non-uniform distribution of corrosion products around rebar.

The simulated time to depassivation shows that the assumption of uniformly distributed corrosion is a strong simplification. For the analyzed test, the time to first local depassivation is three to four years, depending on the chloride threshold value. The depassivation of the whole external rebar surface takes 8.5 to 11 years from the beginning of the initiation phase. In the case of internal rebar, the time to depassivation is significantly extended, and the first points become electro-chemically active after 5.5 to 7.5 years. However, the whole surface is depassivated after 15 to over 20 years. Thus, not only the non-uniform distribution around a rebar, but also the location of particular bars should be included in the calculations of the mechanical problem caused by corrosion.

Moreover, a comparison between the diffusion–migration and the diffusion model is performed to visualize the difference between them. The proposed diffusion–migration model is governed mainly by the diffusion coefficient. It seems impossible to find an exact solution without any experimental data. However, the previous test presented in [5] revealed that, although the model assumes the value of diffusion coefficient which is constant in time, it has the potential to give reliable results of chloride concentration after a long time (e.g., 20 years) knowing the initial value of $D_{cl}$. The parametric studies have been limited to the dependence of the initiation time on the diffusion coefficient and concrete cover size. The chloride threshold value and diffusion coefficient are material parameters different for each concrete composition. The decrease of those two parameters results in an increase of time to depassivation, although the diffusion coefficient value is crucial in this aspect. Another significant parameter is the concrete cover thickness. The thicker the cover is, the longer the initiation phase. However, it must be noticed that preparing a concrete mixture satisfying the condition of a low diffusion coefficient can generate the high cost of a structure. On the other hand, increasing the cover thickness can cause a decrease in load-carrying capacity or increase the dimensions of the element. The choice should be taken by the structure designer.

## Figures and Tables

**Table 1:** Results for 0.3% chloride threshold after 20 years of exposure and concrete cover 50 mm.

Point	External Rebar [Years (Days) to Depassivation]	Internal Rebar [Years (Days) to Depassivation]
S	4.16 (1517)	5.38 (1965)
SW	3.49 (1274)	5.38 (1964)
W	4.16 (1517)	9.90 (3614)
NW	4.85 (1769)	13.60 (4964)
N	7.19 (2626)	14.16 (5167)
NE	8.61 (3143)	14.73 (5376)
E	7.19 (2626)	10.18 (3717)
SE	4.85 (1769)	5.39 (1966)

**Table 2:** Results for 0.35% chloride threshold after 20 years of exposure and concrete cover 50 mm.

Point	External Rebar [Years (Days) to Depassivation]	Internal Rebar [Years (Days) to Depassivation]
S	4.58 (1673)	6.26 (2286)
SW	3.83 (1398)	6.25 (2282)
W	4.58 (1673)	11.75 (4287)
NW	5.42 (1979)	16.33 (5961)
N	8.05 (2938)	17.28 (6306)
NE	9.69 (3538)	18.38 (6709)
E	8.05 (2938)	12.36 (4513)
SE	5.42 (1979)	6.27 (2288)

**Table 3:** Results for 0.4% chloride threshold after 20 years of exposure and concrete cover 50 mm.

Point	External Rebar [Years (Days) to Depassivation]	Internal Rebar [Years (Days) to Depassivation]
S	5.05 (1843)	7.34 (2679)
SW	4.19 (1531)	7.31 (2668)
W	5.05 (1843)	14.13 (5157)
NW	6.06 (2211)	20 (7300)
N	9.04 (3299)	20 (7300)
NE	10.99 (4010)	-
E	9.04 (3299)	15.41 (5625)
SE	6.06 (2211)	7.36 (2685)

## Data Availability

Not applicable.

## Abbreviations

The following abbreviations are used in this manuscript:

RC	reinforced concrete
pH	potential of hydrogen
1D	one-dimensional
CA	cellular automata
FDM	finite difference method
FEM	finite element method

## References

[1] Zybura A., Jaśniok M., Jaśniok T. (2011). Diagnostics of Reinforced Concrete Structures.

[2] Martin-Perez B. (1999). Service Life Modelling of RC Highway Structures Exposed to Chlorides. Ph.D. Dissertation.

[3] Ozbolt J., Balabanic G., Kuster M. (2011). 3D Numerical modelling of steel corrosion in concrete structures. Corros. Sci..

[4] Pantazopoulou S., Papoulia K. (2001). Modelling cover-cracking due to reinforced corrosion in RC structures. J. Eng. Mech..

[5] German M. (2016). Modelling of Chloride Corrosion and Resultant Fracture in RC Elements. Ph.D. Dissertation.

[6] Babaee M., Castel A. (2018). Chloride diffusivity, chloride threshold, and corrosion initiation in reinforced alkali-activated mortars: Role of calcium, alkali, and silicate content. Cem. Concr. Res..

[7] Chalhoub C., François R., Carcasses M. (2019). Determination of chloride threshold initiating corrosion: A new set-up taking the localized aspect of corrosion into account. Cem. Concr. Res..

[8] Van Belleghem B., Kessler S., Van den Heede P., Van Tittelboom K., De Belie N. (2018). Chloride induced reinforcement corrosion behavior in self-healing concrete with encapsulated polyurethane. Cem. Concr. Res..

[9] De Weerdt K., Lothenbach B., Geiker M. (2019). Comparing chloride ingress from seawater and NaCl solution in Portland cement mortar. Cem. Concr. Res..

[10] Song D., Ma A., Sun W., Jiang J., Jiang J., Yang D., Guo G. (2014). Improved corrosion resistance in simulated concrete pore solution of surface nanocrystallized rebar fabricated by wire-brushing. Corros. Sci..

[11] Kawaai K., Nishida T., Saito A., Ujike I., Fujioka S. (2019). Corrosion resistance of steel bars in mortar mixtures mixed with organic matter, microbial or other. Cem. Concr. Res..

[12] Krykowski T., Jaśniok T., Recha F., Karolak M. (2020). A Cracking Model for Reinforced Concrete Cover, Taking Account of the Accumulation of Corrosion Products in the ITZ Layer, and Including Computational and Experimental Verification. Materials.

[13] Chen J., Zhang W., Tang Z., Huang Q. (2020). Experimental and numerical investigation of chloride-induced reinforcement corrosion and mortar cover cracking. Cem. Concr. Compos..

[14] Kenny A., Katz A. (2020). Steel-concrete interface influence on chloride threshold for corrosion—Empirical reinforcement to theory. Constr. Build. Mater..

[15] van Zijl G., Paul S. (2018). A novel link of the time scale in accelerated chloride-induced corrosion test in reinforced SHCC. Constr. Build. Mater..

[16] da Costa A., Fenaux M., Fernández J., Sánchez E., Moragues A. (2013). Modelling of chloride penetration into non-saturated concrete: Case study application for real marine offshore structures. Constr. Build. Mater..

[17] Zhang Y., Ye G. (2019). A model for predicting the relative chloride diffusion coefficient in unsaturated cementitious materials. Cem. Concr. Res..

[18] Tian Y., Chen C., Jin N., Jin X., Tian Z., Yan D., Yu W. (2019). An investigation on the three-dimensional transport of chloride ions in concrete based on X-ray computed tomography technology. Constr. Build. Mater..

[19] Melchers R. (2021). Experience-Based Physico-Chemical Models for Long-Term Reinforcement Corrosion. Corros. Mater. Degrad..

[20] Xia J., Li T., Fang J.X., Jin W.L. (2019). Numerical simulation of steel corrosion in chloride contaminated concrete. Constr. Build. Mater..

[21] Muthulingam S., Rao B. (2014). Non-uniform time-to-corrosion initiation in steel reinforced concrete under chloride environment. Corros. Sci..

[22] Ozbolt J., Balabanic G., Periskic G., Kuster M. (2010). Modelling the effect of damage on transport processes in concrete. Constr. Build. Mater..

[23] Guzman S., Galvez J., Sancho J. (2014). Modelling of chloride ingress into concrete through a single-ion approach. Application to an idealized surface crack pattern. Int. J. Numer. Anal. Methods Geomech..

[24] Samson E., Marchand J., Snyder K., Beaudoin J. (2005). Modeling ion and fluid transport in unsaturated cement systems in isothermal conditions. Cem. Concr. Res..

[25] Krykowski T. (2012). Modeling of Concrete Cover Damage Caused by Rebar Corrosion in Reinforced Concrete.

[26] Guzman S., Galvez J., Sancho J. (2011). Cover cracking of reinforced concrete due to rebar corrosion induced by chloride penetration. Cem. Concr. Res..

[27] Ozbolt J., Orsanic F., Balabanic G. (2012). Modeling damage in concrete caused by corrosion of reinforcement: Coupled 3D FE model. Int. J. Fract..

[28] Ozbolt J., Orsanic F., Balabanic G. (2014). Modeling pull-out resistance of corroded reinforcement in concrete: Coupled three-dimensional finite element model. Cem. Concr. Res..

[29] Angst U. (2019). Predicting the time to corrosion initiation in reinforced concrete structures exposed to chlorides. Cem. Concr. Res..

[30] Liu Y. (1996). Modeling the Time-to-Corrosion Cracking of the Cover Concrete in Chloride Contaminated Reinforced Concrete Structures. Ph.D. Thesis.

[31] Balafas I., Burgoyne C. (2010). Environmental effects on cover cracking due to corrosion. Cem. Concr. Res..

[32] Geiker M., Danner T., Michel A., Belda Revert A., Linderoth O., Hornbostel K. (2021). 25 years of field exposure of pre-cracked concrete beams: Combined impact of spacers and cracks on reinforcement corrosion. Constr. Build. Mater..

[33] Michel A., Sørensen H., Geiker M. (2021). 5 years of in situ reinforcement corrosion monitoring in the splash and submerged zone of a cracked concrete element. Constr. Build. Mater..

[34] Fu C., Ye H., Jin X., Yan D., Jin N., Peng Z. (2016). Chloride penetration into concrete damaged by uniaxial tensile fatigue loading. Constr. Build. Mater..

[35] Bezuidenhout S., van Zijl G. (2019). Corrosion propagation in cracked reinforced concrete, toward determining residual service life. Struct. Concr..

[36] Chen F., Zhong Y., Gao X., Jin Z., Wang E., Zhu F., Shao X., He X. (2021). Non-uniform model of relationship between surface strain and rust expansion force of reinforced concrete. Sci. Rep..

[37] Nielsen E., Geiker M. (2002). Chloride diffusion in partially saturated cementitious material. Cem. Concr. Res..

[38] Biondini F., Bontempi F., Frangopol D., Malerba P. (2004). Cellular automata approach to durability analysis of concrete structures in aggressive environments. J. Struct. Eng..

[39] Oh B., Jang S. (2007). Effects of material and environmental parameters on chloride penetration. Cem. Concr. Res..

[40] Samson E., Marchand J., Beaudoin J. (1999). Describing ion diffusion mechanisms in cement-based materials using the homogenization technique. Cem. Concr. Res..

[41] Liu T., Weyers R. (1998). Modeling the dynamic corrosion process in chloride contaminated concrete structures. Cem. Concr. Res..

[42] Jaśniok T. (2004). Identification of Corrosion Rate of Reinforcement Due to Polarization Measurements. Ph.D. Dissertation.

[43] Krykowski T., Zybura A. (2009). FEM modelling of concrete degradation caused by rebar corrosion in reinforced concrete. Archit. Civ. Eng. Environ..

[44] Pluciński P. (2008). Numerical Analysis of Mechanical Effects of Rebar Corrosion in Concrete Structures. Ph.D. Dissertation.

[45] Zaborski A. (2007). Concrete elements durability in aggressive environments: Cellular automata simulation. Environmental Effects on Buildings, Structures, Materials and People.

[46] Thomas M., Bamforth P. (1999). Modelling chloride diffusion in concrete. Effect of fly ash. Cem. Concr. Res..

[47] Sandberg P. (1998). Chloride Initiated Reinforcement Corrosion in Marine Concrete.

[48] German M., Pamin J. (2015). FEM simulations of cracking in RC beams due to corrosion progress. Arch. Civ. Mech. Eng..

[49] Bazant Z. (1979). Physical model for steel corrosion in concrete sea structures–Theory. J. Struct. Div..

